# Body mass index, body fat percentage, and visceral fat as mediators in the association between health literacy and hypertension among residents living in rural and suburban areas

**DOI:** 10.3389/fmed.2022.877013

**Published:** 2022-09-06

**Authors:** Tham T. Nguyen, Minh H. Nguyen, Yen H. Nguyen, Thao T. P. Nguyen, Manh H. Giap, Tung D. X. Tran, Thu T. M. Pham, Khue M. Pham, Kien T. Nguyen, Vinh-Tuyen T. Le, Chien-Tien Su, Tuyen Van Duong

**Affiliations:** ^1^Faculty of Public Health, Hai Phong University of Medicine and Pharmacy, Hai Phong, Vietnam; ^2^International Ph.D. Program in Medicine, College of Medicine, Taipei Medical University, Taipei, Taiwan; ^3^Department of Pharmacology and Clinical Pharmacy, Can Tho University of Medicine and Pharmacy, Can Tho, Vietnam; ^4^Department of Pharmacy, Can Tho University of Medicine and Pharmacy Hospital, Can Tho, Vietnam; ^5^Ph.D. Program in School of Pharmacy, College of Pharmacy, Taipei Medical University, Taipei, Taiwan; ^6^Health Personnel Training Institute, University of Medicine and Pharmacy, Hue University, Hue, Vietnam; ^7^Emergency Department, Bai Chay Hospital, Hạ Long, Vietnam; ^8^Stem Cell Unit, Van Hanh Hospital, Ho Chi Minh, Vietnam; ^9^Hi-Tech Institute, Nguyen Tat Thanh University, Ho Chi Minh, Vietnam; ^10^School of Public Health, College of Public Health, Taipei Medical University, Taipei, Taiwan; ^11^Department of Health Promotion, Faculty of Social and Behavioral Sciences, Hanoi University of Public Health, Hanoi, Vietnam; ^12^Department of Pharmacognosy - Traditional Pharmacy - Pharmaceutical Botanic, Can Tho University of Medicine and Pharmacy, Can Tho, Vietnam; ^13^Ph.D. Program in Clinical Drug Development of Herbal Medicine, College of Pharmacy, Taipei Medical University, Taipei, Taiwan; ^14^School of Public Health, Taipei Medical University, Taipei, Taiwan; ^15^Department of Family Medicine, Taipei Medical University Hospital, Taipei, Taiwan; ^16^School of Nutrition and Health Sciences, Taipei Medical University, Taipei, Taiwan

**Keywords:** health literacy, hypertension, body fat percentage, visceral fat, body mass index, rural people

## Abstract

**Background:**

Hypertension is a major cause of death and disability worldwide. Enhancing health literacy (HL) may help to alleviate the risk of hypertension and its burden. However, evidence on the association between HL and hypertension and potential mechanisms remain to be explored.

**Objectives:**

This study examined the association between HL and hypertension; and explored whether body mass index (BMI), body fat percentage (PBF), and visceral fat (VF) were mediators of this association in people who resided in rural and suburban areas in Vietnam.

**Methods:**

A cross-sectional survey was conducted from 1st July to 31st December 2019, involving 1655 residents and exploring participants' sociodemographic characteristics, HL, health-related behaviors, comorbidities, body composition, and blood pressure (BP). People with systolic BP ≥ 140 mmHg or diastolic BP ≥ 90 mmHg or using antihypertensive medication were classified as having hypertension. Multiple logistic regression and mediation analyses were used to explore associations.

**Results:**

The hypertension prevalence was 41.9% (694/1,655). In adjusted models, a higher HL score was associated with a lower hypertension likelihood (OR = 0.96; 95%CI = 0.95–0.97; *p* < 0.001). Factors associated with a higher odd of hypertension were overweight/obese (OR = 1.69; 95%CI = 1.24–2.29; *p* = 0.001), high PBF (OR = 2.35; 95%CI = 1.85–2.99; *p* < 0.001), and high VF (OR = 2.27; 95%CI = 1.63–3.16; *p* < 0.001). Notably, PBF significantly mediated the association between HL and hypertension (indirect effect, OR = 0.99; 95%CI = 0.98–0.99; *p* = 0.009; percent mediated = 8.56%). The mediating roles of BMI and VF were not found.

**Conclusion:**

The prevalence of hypertension was relatively high. People with better HL were less likely to have hypertension. The association between HL and hypertension was partially explained by PBF. Strategic approaches are required to improve people's HL and body fat which further help to manage hypertension in rural and suburban areas.

## Introduction

Globally, cardiovascular diseases (CVDs) are the leading cause of death, accounting for 32% (over 17 million) of all deaths (1). The main risk factor of CVDs is elevated blood pressure or hypertension, which is estimated to cause 8.5 million deaths worldwide in 2015 (2, 3). Approximately 1.28 billion people in the age group of 30 to 79 years have hypertension, of which two-thirds come from low- and middle-income countries (LMICs) (4). Furthermore, the overall prevalence of hypertension in LMICs (31.5%) was higher than in high-income countries (HICs) (28.5%) (5). Therefore, the burden of hypertension is transiting from developed countries to LMICs (6).

Vietnam is a developing country in the process of socio-economic reform. As a result, the disease pattern gradually shifts from infectious diseases to non-communicable diseases (7, 8). A recent meta-analysis study showed that the pooled prevalence of hypertension in Vietnam was 18.5% according to three national surveys and 21.1% as reported in ten studies (9). In addition, hypertension rates vary by race and geography (10, 11).

According to a census in 2019, the number of people living in rural areas in Vietnam was 63 million, accounting for 65.4% of the total population (12). Rural areas are also home to different ethnic minorities in Vietnam. In the period of industrialization and modernization, population aging and rapid economic development have been causing a transition in the epidemiology of diseases in rural Vietnam. Thus, the prevalence and risk factors of hypertension also change. Furthermore, in some rural regions, medical facilities are inadequately equipped and difficult to access, leading to a high rate of uncontrolled hypertension (6, 13). Therefore, it is essential to assess the status and influencing factors of HTN in rural areas, which could help to develop effective strategies to improve the detection and management of hypertension, reducing its burden.

Positive changes in health behaviors and management of hypertension play a crucial role in alleviating the risk and burden of hypertension (6, 14). Health literacy (HL) defined as “the ability to find, evaluate, understand and apply health information to make proper health decisions” also showed its important role (15). Sufficient HL was associated with improved health-related behaviors (treatment adherence, healthier diet, staying physically active) (16–18) and a lower risk of obesity (19, 20). Notably, the Institute of Medicine in the US also emphasized the importance of additional research to evaluate and better understand the role of HL in clinical outcomes (21). However, the relationship between HL and hypertension and its mechanism has not been adequately studied. A previous study conducted in a multi-racial population in the Netherlands indicated that HL was associated with a lower likelihood of hypertension in some races (22). Other studies in hospitalized or dialysis patients have also shown that HL has an inverse association with blood pressure (23, 24). However, the HL level among Vietnamese people, especially in rural areas, is relatively low (25, 26). Thus, assessing the relationship between health literacy and hypertension is essential, which could provide evidence for interventions to enhance HL for the rural population, thereby helping to reduce the prevalence of hypertension and its burden.

Previous studies have shown that several factors such as high salt consumption, alcohol consumption, smoking, physical inactivity, imbalanced diet, and obesity significantly increase the risk of hypertension (27, 28). Among those risks, obesity is a global public health challenge, with the number of people over 18 years old being overweight and obese in 2016 at 39 and 13%, respectively (29). The prevalence of obesity tends to increase faster in LMICs than in developed countries that exacerbated the risk and burden of obesity-related NCDs in the LMICs (30). With rapid economic development, rural areas have also undergone a shift in traditional eating patterns, resulting in changes in body composition and the obesity rate. However, research on the relationship between body composition (visceral fat, body fat percentage) and hypertension in rural populations is scarce.

Therefore, we conducted this study to (1) examine the association between health literacy and hypertension and (2) explore mediating roles of BMI, abdominal obesity, body fat percentage, and visceral fat among residents living in rural and suburban areas in Vietnam.

## Materials and methods

### Study design and sampling

We conducted a cross-sectional survey from 1st July to 31st December 2019, in four cities and provinces of Vietnam in conjunction with free medical examination trips organized by hospitals and medical universities, including two in the north (Hai Phong city organized by Haiphong University of Medicine and Pharmacy, Quang Ninh province organized by Bai Chay Hospital), one in the center (Thua Thien Hue province organized by University of Medicine and Pharmacy, Hue University), and one in the south (Lam Dong province organized by Van Hanh Hospital). These medical trips were conducted at community health stations in rural and suburban areas.

Participants were consecutively selected when they visited community health stations for free medical examination. Residents recruited were those (1) who lived in rural and suburban areas in four selected provinces, (2) aged over 18 years old, (3) can understand the Vietnamese language or local dialect. People with diagnosed mental disorders, or communication difficulties (such as deafness), or any emergency conditions (e.g., severe trauma, difficulty breathing, stroke, acute inflammations) were excluded from the study.

Finally, a total of 1,655 people were interviewed, their data were analyzed, including 440 from Thua Thien Hue province, 586 from Quang Ninh province, 503 from Hai Phong city, and 126 from Lam Dong province.

### Data collection procedure

The interviewers (including medical doctors, nurses, and medical students) had received a 4-h training about data collection conducted by senior researchers. A meeting between the research team, the medical team, and the local volunteers was conducted to plan and arrange the medical examination and survey interview properly. The face-to-face interviews were conducted at community health stations using printed questionnaires. Local volunteers helped interviewers with translating and communicating with people who spoke the local dialect. Participants were asked to sign an informed consent form before their participation. Each interview took about 15–20 min. The completed questionnaires were re-checked by senior researchers to ensure that missing data was minimal. Obtained data was cleaned and analyzed confidentially for research purposes.

### Ethical consideration

The study was approved by the Institutional Review Board of Hanoi University of Public Health (IRB number: 379/2019/YTCC-HD3 and 479/2019/YTCC-HD3).

### Measurements

#### Sociodemographic characteristics

The data collected includes participant's characteristics regarding age (year), gender (women vs. men), marital status (single vs. married, separated/divorced/widowed), education levels (junior high school or below vs. senior high school, college/university or above), employment status (unemployed vs. employed), ability to pay for healthcare (easy vs. difficult), social status (low vs. middle or high). The Kinh people is the most predominant ethnic that accounts for over 85.32% of the population of Vietnam according to the 2019 Census (12). Therefore, we dichotomized the ethnicity into 2 levels: “Kinh” vs. “Ethnic minorities.” Participants were also asked about whether they were currently diagnosed by a doctor with one of the following medical conditions, including diabetes, cardiovascular disease, liver disease, cancer, cerebrovascular disease, lung disease, kidney disease, mental illness, arthritis, eye disease, ear nose throat (ENT) diseases. Medical conditions were classified into two groups: “none” vs. “one or more.”

#### Hypertension

Blood pressure (BP, mmHg), including systolic (SBP) and diastolic BP (DBP), were measured using a standard clinical manual aneroid sphygmomanometer by health professionals. First, the medical staff asked participants if they were feeling anxious when they sat down to have their blood pressure measured. If so, they were asked to rest for 10–15 min to calm down before taking their BP. Next, we measured BP three times in the right arm in a sitting position. The interval between each measurement is 30 s. The average values of SBP and DBP in three measurements were recorded. We also investigated whether participants had been diagnosed with hypertension and treated with antihypertensive drugs. Hypertension was classified if people had SBP of ≥140 mmHg and/or DBP of ≥90 mmHg (31) or were using antihypertensive medications.

#### Body composition and anthropometric parameters

Bodyweight (kg), height (cm), waist (cm), and hip circumference (cm) were measured when participants were wearing light clothing and no shoes, using a weighing scale and tape measure with the precision of 0.1 kg and 0.1 cm, respectively. The body mass index (BMI, kg/m^2^) was calculated from body weight and height and categorized into three groups, including underweight (BMI <18.5), normal weight (18.5 ≤ BMI < 23), and overweight or obese (BMI ≥ 23) (32, 33). Abdominal obesity (no vs. yes) was classified based on the waist-hip ratio of ≥0.90 for males and ≥0.85 for females (34).

The body composition, including body fat percentage (PBF) and visceral fat (VF), were measured using portable bioelectrical impedance analysis devices (InBody H20B, Seoul, South Korea) by doctors or nurses according to the manufacturer's instructions. We classified VF into two groups, including normal (VF ≤ 9) and high (VF > 9) (35, 36). PBF was categorized into three groups, including low (<10% for men and <20% for women), normal (10–19% for men and 20–29% for women), and high (≥20 for men and ≥30 for women) (36–38).

#### Health literacy

We used the 12-item short-form health literacy (HLS-SF12) questionnaire to evaluate the health literacy of rural people. This instrument was validated in the Vietnamese rural population (25) and extensively used in many studies (39–43). In this study, the Cronbach's alpha of HLS-SF12 was 0.91. Participants were asked to rate their ability to conduct each item with four responses options from 1 = “very difficult” to 4 = “very easy.” The HL index was calculated using a formula:


(1) $$\begin{array}{r} \text{Index}=\left(\text{mean}-1\right)\times \left(50/3\right)\;\left(1\right) \end{array}$$

Where *Index* is a new standardized score of HL, *mean* is the mean of 12 items for each respondent, 1 is the minimum value of the mean, 3 is the mean range, and 50 is the selected maximum value of the new metric. The HL index scores ranged from 0 to 50, with a higher score indicating better HL (25, 26).

#### Health-related behaviors

Participants were asked about cigarette smoking (none, used to, current), alcohol drinking (none, 1–3 times per month, 1–5 times per week, every day), physical activity (none, sometimes, often, every day). Eating behaviors were assessed using a 5-item healthy eating score (HES-5) questionnaire (44, 45). The HES-5 questionnaire was validated and used in different populations in Vietnam (46–48). Participants were investigated about the frequency of consuming the five following foods, including fruits, vegetables, fish, whole grains, and dairy products, with six possible responses (from 0 = “rarely or never” to 5 = “more than two times per day”). The range of HES-5 score is from 0 to 25, where people with a higher score had a healthier diet.

Salt-related knowledge was evaluated using the Short Sodium Knowledge Survey (SSKS) (49) with three questions related to identifying (1) higher salt-containing foods, (2) source of salt in meals, and (3) a way to lower salt intake. All correct responses were assigned a score of one, while wrong responses and non-responses were scored as zero. The sum scores were between 0 and 3, with higher scores indicating better knowledge about dietary salt.

Salt-related behaviors were assessed using four questions from the “Knowledge, Attitudes, and Behavior toward Dietary Salt” questionnaire developed by the world health organization, or WHO (50). The first three questions assessed the frequency of (1) consumption of high-salt processed foods and the addition of salt in (2) cooking and (3) eating. Participants responded with five frequency levels from 1 = “Always” to 5 = “Never.” The fourth question evaluated participant's perception of their salt consumption with five answer options from 1 = “far too much” to 5 = “far too little.” The sum scores ranged from 0 to 20, with higher scores indicating better salt-related behaviors.

### Data analysis

First, the frequency, percentage, mean, and standard deviation of participant's characteristics were reported appropriately. Next, we conducted the Chi-squared test to compare the proportions of hypertension in different groups of independent variables (IVs). Then, we used simple and adjusted logistic regression models to explore the associations of HL, BMI, abdominal obesity, VF, and PBF with hypertension. Age, sex, and IVs associated with hypertension at *p*-value <0.2 in simple regression models were adjusted in final models (Supplementary Table S1). We performed the Spearman correlation test to explore relationships between IVs to eliminate multicollinearity. If two factors had a moderate or high correlation (rho ≥ 0.3), a representative one was chosen to adjust the final models (Supplementary Table S2). Finally, we conducted mediation analyses using the *khb* (Karlson Holm Breen) method to explore the degree to which BMI, VF, and PBF may explain the association between HL and hypertension. The *khb* method can be used for logistic regression models to decompose the total effect of HL on hypertension (not adjusted for the mediating factor) into the direct effect (the impact of HL on hypertension adjusted for the mediating factor) and indirect effects (the mediating effect) (51). Each potential mediator was tested in separate models. The mediated percentage was also calculated as the ratio of the indirect effect to the total effect. If the mediation analysis was statistically significant, a mediation model was designed to illustrate the relationships between HL, the mediating factor, and hypertension. The *p*-value < 0.05 was considered significant. For data analysis, we used Stata for Windows, version 15.1 (StataCorp LLC, College Station, TX, USA).

## Results

### Participant's characteristics

In this study, the average age of participants was 50.1 ± 16.7. Of all the sample, 64.2% (1,061/1,655) were female, 46.2% (764/1,655) were from ethnic minorities, 69.8% (1,155/1,655) had a junior high school degree or below, 92.7% (1,518/1,655) were ever married, 23.1% (383/1,655) were unemployed, 64.1% (1,056/1,655) found it difficult to pay for healthcare, 49.0% (811/1,655) had at least one disease, 14.3% (235/1655) were overweight or obese, and 50.9 (842/1,655) had abdominal obesity. The mean scores of health literacy, HES, sodium knowledge, and salt intake practice were 22.1 ± 11.2, 9.5 ± 4.5, 1.2 ± 0.8, and 11.2 ± 2.0, respectively. Regarding body composition, the average values of PBF and VF were 28.4 ± 10.9 and 5.6 ± 4.7, respectively, of which 12.5% (207/1,655) had high VF, 8.8% (145/1,655) had low PBF, and 53.1% (873/1,655) had high PBF. The prevalence of hypertension in the current study was 41.9% (694/1,655), in which 29% (481/1,655) did not know that they had hypertension or have been not diagnosed previously, and 12.9% (213/1,655) participants declared to take antihypertensive medications. The proportion of hypertension was varied by different categories of age, gender, marital status, education, occupation, ability to pay for healthcare, social status, comorbidities, smoking, drinking, BMI, VF, and PBF (Table 1).

**Table 1:** Characteristics of participants (*n* = 1,655).

**Variables**	**Total (*n* = 1,655)**	**Non-HTN (*n* = 961)**	**HTN (*n* = 694)**	
	***n* (%)**	***n* (%)**	***n* (%)**	***p* ^a^**
Age (years), mean ± SD	50.1 ± 16.7	45.1 ± 15.7	57.1 ± 15.4	
**Age groups**				<0.001
<60	1,141 (68.9)	767 (79.8)	374 (53.9)	
≥60	514 (31.1)	194 (20.2)	320 (46.1)	
**Gender**				<0.001
Women	1,061 (64.1)	669 (69.7)	392 (56.7)	
Men	591 (35.7)	291 (30.3)	300 (43.3)	
**Ethnicity**				0.871
Kinh	891 (53.8)	519 (54.0)	372 (53.6)	
Ethnic minorities	764 (46.2)	442 (46.0)	322 (46.4)	
**Marital status**				<0.001
Single	119 (7.2)	81 (8.4)	38 (5.5)	
Married	1,378 (83.3)	821 (85.4)	557 (80.2)	
Separated/Divorced/Widowed	140 (8.5)	54 (5.6)	86 (12.4)	
**Education attainment**				<0.001
Junior high school or below	1,155 (69.8)	605 (62.9)	550 (79.2)	
Senior high school	268 (16.2)	188 (19.5)	80 (11.5)	
College/University or above	232 (14.0)	168 (17.5)	64 (9.2)	
**Employment status**				<0.001
Unemployed	383 (23.1)	165 (17.2)	218 (31.4)	
Employed	1,272 (76.9)	796 (82.8)	476 (68.6)	
**Ability to pay for healthcare**				<0.001
Very or fairly easy	591 (35.7)	398 (41.4)	193 (27.8)	
Very or fairly difficult	1,056 (63.8)	559 (58.2)	497 (71.6)	
**Social status**				<0.001
Low	480 (29.0)	244 (25.4)	236 (34.0)	
Middle or high	1,166 (70.5)	711 (73.9)	455 (65.6)	
**Medical conditions**				<0.001
None	843 (51.0)	552 (57.4)	291 (41.9)	
One or more	811 (49.0)	409 (42.5)	402 (57.9)	
**Cigarette smoking**				0.031
None	1,299 (78.5)	773 (80.4)	526 (75.8)	
Used to	52 (3.1)	23 (2.4)	29 (4.2)	
Current	302 (18.3)	164 (17.0)	138 (19.9)	
**Drinking alcohol**				<0.001
None	1,120 (67.7)	673 (70.0)	447 (64.4)	
1–3 times/month	203 (12.3)	130 (13.5)	73 (10.5)	
1–5 times/week	239 (14.5)	120 (12.5)	119 (17.2)	
Everyday	90 (5.4)	37 (3.9)	53 (7.6)	
**Physical activity**				0.114
None	685 (41.4)	405 (42.1)	280 (40.3)	
Sometimes	175 (10.6)	110 (11.4)	65 (9.4)	
Often	322 (19.5)	192 (19.9)	130 (18.7)	
Everyday	471 (28.5)	253 (26.3)	218 (31.4)	
**BMI, kg/m** ^**2**^				<0.001
Underweight	204 (12.3)	125 (13.0)	79 (11.4)	
Normal weight	929 (56.1)	585 (60.8)	344 (49.6)	
Overweight/obese	512 (30.9)	248 (25.8)	264 (38.0)	
**Abdominal obesity**				0.614
No	813 (49.1)	467 (48.6)	346 (49.8)	
Yes	842 (50.9)	494 (51.4)	348 (50.2)	
**Visceral fat level**				<0.001
Normal	1,445 (87.3)	879 (91.5)	566 (81.5)	
High	207 (12.5)	81 (8.4)	126 (18.2)	
**Body fat percentage**				<0.001
Low	145 (8.8)	101 (10.5)	44 (6.3)	
Normal	625 (38.0)	440 (45.8)	185 (16.7)	
High	873 (53.1)	415 (43.2)	458 (66.0)	
**Hypertension**				
No	961 (58.1)			
Yes	694 (41.9)			
Healthy eating score, mean ± SD	9.5 ± 4.5	9.7 ± 4.5	9.4 ± 4.5	0.148
Salt-related knowledge, mean ± SD	1.2 ± 0.8	1.2 ± 0.8	1.1 ± 0.8	0.201
Salt-related behaviors, mean ± SD	11.2 ± 2.0	11.3 ± 1.9	11.0 ± 2.1	0.286
Health literacy, mean ± SD	22.1 ± 11.2	24.2 ± 11.2	19.1 ± 10.4	<0.001
WHR, %, mean ± SD	0.8 ± 0.1	0.8 ± 0.1	0.9 ± 0.1	<0.001
PBF, %, mean ± SD	28.4 ± 10.9	27.2 ± 10.4	30.1 ± 11.5	<0.001
VF, mean ± SD	5.6 ± 4.7	5.3 ± 4.5	6.1 ± 5.0	0.001
SBP, mmHg, mean ± SD	123.9 ± 23.2	–	–	–
DBP, mmHg, mean ± SD	73.7 ± 13.8	–	–	–

HTN, hypertension; SD, standard deviation; WHR, waist-hip ratio; VF, visceral fat level; PBF, body fat percentage; SBP, systolic blood pressure; DBP, diastolic blood pressure.

^a^The p-value of the Chi-square or one-way ANOVA test appropriately.

### Associations of health literacy, BMI, abdominal obesity, visceral fat level, and body fat percentage with hypertension

In simple logistic regression models, age, gender, marital status, education, occupation, ability to pay for healthcare, social status, medical conditions, smoking, drinking, physical activity, and HES were associated with hypertension at *p* <0.2 (Supplementary Table S1). However, after checking Spearman's correlations between these factors, we found that age was moderately correlated with occupation (*rho* = −0.418), gender was moderately correlated with smoking (*rho* = 0.415) and drinking (*rho* = 0.505), education was moderately correlated with marital status (*rho* = −0.303) and ability to pay for healthcare (*rho* = −0.359) (Supplementary Table S2). Therefore, age, gender, education, social status, medical conditions, physical activity, and HES were adjusted in the final models.

After adjusting for confounders, the results showed that higher health literacy scores were associated with a lower likelihood of having hypertension (odds ratio, OR = 0.96; 95% confidence interval, 95% CI = 0.95–0.97; *p* < 0.001). In contrast, respondents who were overweight or obese (OR = 1.94; 95% CI = 1.52–2.47; *p* < 0.001), or who had a high VF (OR = 2.27; 95% CI = 1.63–3.16; *p* < 0.001), or who had a high PBF (OR = 2.35; 95% CI = 1.85–2.99; *p* < 0.001) were more likely to have hypertension (Table 2).

**Table 2:** Associations of BMI, abdominal obesity, body fat percentage, visceral fat level, and health literacy with hypertension (*n* = 1,655).

**Variables^b^**	**Hypertension**			
	**OR (95% CI)**	***p***	**OR (95% CI) ^a^**	***p***
Health literacy, 1-score increment	0.96 (0.95, 0.98)	<0.001	0.96 (0.95, 0.97)	<0.001
**BMI, kg/m** ^**2**^				
Underweight	0.92 (0.68, 1.25)	0.608	0.85 (0.61, 1.20)	0.368
Normal weight	1.00		1.00	
Overweight/obese	1.47 (1.11, 1.95)	0.007	1.94 (1.52, 2.47)	<0.001
**Abdominal obesity**				
No	1.00		1.00	
Yes	1.03 (0.83, 1.29)	0.770	1.02 (0.82, 1.27)	0.856
**Visceral fat level**				
Normal	1.00		1.00	
High	2.42 (1.79, 3.25)	<0.001	2.27 (1.63, 3.16)	<0.001
**Body fat percentage**				
Low	1.04 (0.69, 1.54)	0.860	1.05 (0.69, 1.60)	0.822
Normal	1.00		1.00	
High	2.62 (2.11, 3.26)	<0.001	2.35 (1.85, 2.99)	<0.001

OR, odds ratio; CI, confidence interval; VF, visceral fat level.

^a^Adjusted for age, gender, education, social status, medical conditions, physical activity, healthy eating score, and health literacy.

^b^BMI, abdominal obesity, visceral fat level, and body fat percentage were analyzed in separate models.

### Mediation analyses of body mass index, visceral fat level, body fat percentage on the association between health literacy and hypertension

**Figure 1:**
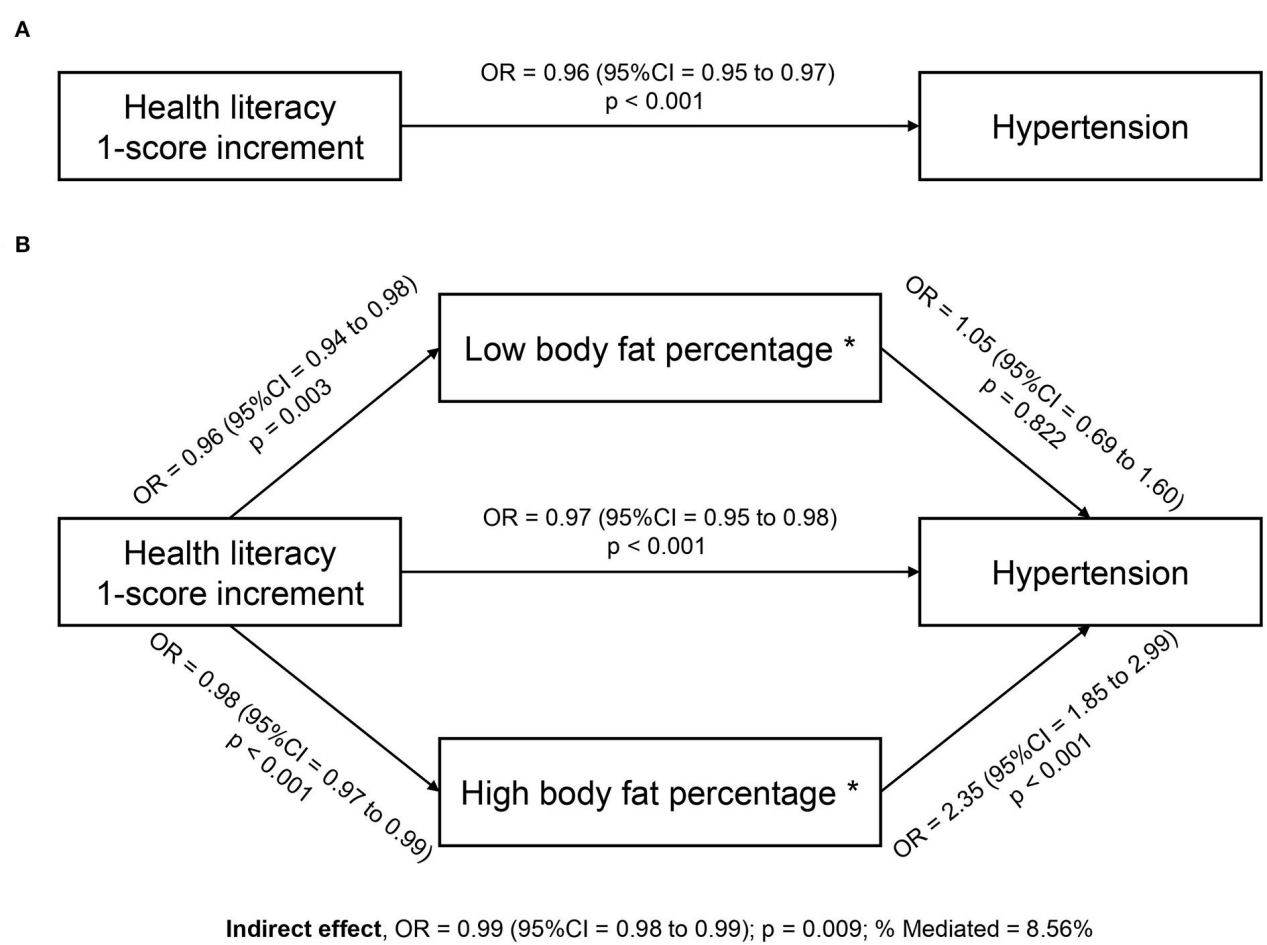
Body fat percentage mediates the association between health literacy and hypertension among rural people (*n* = 1,655). *The reference group is normal body fat percentage. **(A)** Total effect; **(B)** Direct and indirect effects. Mediation models were adjusted for age, gender, education, social status, medical conditions, physical activity, and healthy eating score.

We used the *khb* method to examine the extent to which the association between health literacy and hypertension can be explained by BMI, VF, and PBF. The results of mediation analyses indicated that only PBF partially mediated the association between health literacy and hypertension. This association was explained 8.56% by PBF (Table 3). The mediation role of PBF was illustrated in Figure 1. The total effect of the association without a mediator was significant (OR = 0.96; 95% CI = 0.95–0.97; *p* < 0.001) (Figure 1A). With PBF as a mediator, the direct effect of the association remained significant and was increased (OR = 0.97; 95% CI = 0.95–0.98; *p* < 0.001). In the first paths of indirect effects, higher health literacy was associated with lower likelihoods of having low PBF (OR = 0.96; 95% CI = 0.94–0.98; *p* = 0.003) or high PBF (OR = 0.98; 95% CI = 0.97–0.99; *p* < 0.001). However, in the second paths, only high PBF was associated with a higher likelihood of hypertension (OR = 2.35; 95% CI = 1.85–2.99; *p* < 0.001) (Figure 1B). The effects of two models without and with PBF were significantly different (indirect effect, OR = 0.99; 95% CI = 0.98–0.99; *p* = 0.009) (Table 3). The association was not mediated by VF and BMI.

**Table 3:** Body mass index, body fat percentage, and visceral fat level as mediators in the association between health literacy and hypertension (*n* = 1,655).

**Mediators**	**Effect**	**OR (95% CI)**	***p* ^a^**	**% Mediated^b^**
Body mass index	Total	0.96 (0.95, 0.97)	<0.001	–
	Direct	0.96 (0.94, 0.97)	<0.001	
	Indirect	1.00 (0.99, 1.00)	0.321	
Visceral fat level	Total	0.96 (0.95, 0.97)	<0.001	–
	Direct	0.96 (0.95, 0.97)	<0.001	
	Indirect	0.99 (0.98, 1.00)	0.126	
Body fat percentage	Total	0.96 (0.95, 0.97)	<0.001	8.56%
	Direct	0.97 (0.95, 0.98)	<0.001	
	Indirect	0.99 (0.98, 0.99)	0.009	

OR, odds ratio; CI, confidence interval.

^a^Adjusted for age, gender, education, social status, medical conditions, physical activity, healthy eating score.

^b^The mediated percentage was calculated only when the indirect effect was significant (p < 0.05).

## Discussion

In this study, the prevalence of hypertension was 41.9%. The finding was higher than the results from meta-analyses studies, with the pooled prevalence of hypertension in adults (2005–2018) in Vietnam of 21.1% (9) and East Asia and the Pacific (2001–2015) of 35.5% (52). However, compared with studies conducted in rural populations, our results are consistent with a study in 6 Latin American countries (42.1%) (53) and lower than studies in China (53.6%), Madagascar (49.1%), Pakistan (46.8%), and the northern mountainous area of Vietnam (47.6%) (54–57). The variation in prevalence across studies can be due to differences in measurement tools or diagnostic guidelines. Notably, the average age in this study is relatively high (50.1 ± 16.7) because young people in rural Vietnam or low- or middle-income countries tend to go to big cities to find work. In addition, advanced age is a non-modifiable risk factor for hypertension (28). This may explain why the prevalence of hypertension in this study was much higher than that in the general population in Vietnam. Therefore, health authorities should focus on strengthening the diagnosis, detection, and management of hypertension in rural areas, thereby reducing the burden of morbidity and mortality from hypertension.

The results of this study indicated that overweight or obese people (BMI ≥ 25) were more likely to have hypertension than those with normal weight. This result was in line with previous studies indicating higher BMI was positively associated with blood pressure and the development of hypertension (58–62). Results from a meta-analysis study with 25 randomized clinical trials showed that losing one kilogram in body weight was associated with a 1.05 mmHg and 2.05 mmHg reduction in systolic and diastolic blood pressure, respectively (63). In addition, we found that high VF was linked to a higher likelihood of having hypertension. Visceral adiposity has been proven to be associated with higher blood pressure (64–66). The relationship between visceral fat and hypertension was also documented in other studies (36, 60, 67–69). Furthermore, our findings also showed that high PBF, but not low PBF, was positively associated with the prevalence of hypertension in rural people. A recent study conducted among the rural population in Henan province of China showed that higher quartile groups of PBF were linked to higher odds of having hypertension (70). This association was also in line with prior studies conducted in different populations in Korea, China, and England (69, 71–74). Another research suggested that compared to other adiposity indices such as BMI or waist-hip ratio, PBF and VF were better predictors of hypertension (75). However, as the underlying mechanism of PBF and VF with hypertension is still unclear, further studies are needed to confirm and explain.

Our study found that people with higher health literacy scores were less likely to have hypertension. Research on the relationship between HL and HTN is still limited. A study conducted in a multi-ethnic population in Amsterdam, the Netherlands, indicated that low health literacy was positively associated with hypertension in Dutch and African Surinamese, but not South-Asian Surinamese (22). Several previous studies in hospitalized patients without hypertension and dialysis patients have also shown inadequate HL was associated with increased blood pressure (23, 24). The possible explanation for the relationship between health literacy and hypertension is that people with adequate health literacy were less likely to be overweight or obese (19, 20) or engage in harmful lifestyles, such as unhealthy diets, excessive sodium intake, physical inactivity, smoking, and drinking (17, 18, 76). This can reduce the risk of hypertension. In addition, among those with comorbidities such as diabetes or kidney disease, health literacy was found to have a positive association with treatment adherence (77–80), which may help patients better control blood pressure and reduce the likelihood of developing complications of these diseases, including hypertension.

To our best knowledge, no previous study has suggested an explanatory mechanism for the association between health literacy and hypertension. Therefore, this study explored the mediating role of body composition indicators, including BMI, VF, and PBF, in this relationship. Our findings suggested that only PBF significantly mediated the association between health literacy and hypertension after adjusting for potential confounders and could explain 8.56% of this association. In the mediation pathways, people with a higher health literacy score had lower odds of having low PBF or high PBF. However, only high PBF was positively associated with the likelihood of hypertension. Thus, the role of health literacy in mitigating the likelihood of high PBF may partially explain the observed relationship of health literacy with hypertension. Besides, it is suggested that health-related behaviors (diet, physical activity, salt intake, consumption of cigarettes and alcohol) may be potential mediators of the health literacy and hypertension association. Mediation analyses were also conducted for these factors, but none significantly mediated this association. These results were reported in Supplementary Table S3. Therefore, we used these factors as confounders to adjust in final models.

With a relatively large sample, this study could suggest evidence for health organizations and policymakers to promote appropriate strategies to enhance health literacy for people living in the rural and suburban areas, thereby helping to better prevent and manage hypertension. Furthermore, our findings also contribute a preliminary mechanism to explain the association between health literacy and hypertension, future longitudinal studies with a representative sample should be conducted to validate our results. However, the current research holds several limitations that may weaken the certainty of our results. First, the cause-effect relationship was not established from this cross-sectional study. Second, as the total number of people who visited community health stations during the study period was not recorded, we cannot calculate the response rate. In addition, the sample recruited in this study may not be representative of the whole rural population. Therefore, those may affect the generalizability of our findings. Third, as some factors, such as health-related behaviors, were assessed by the questionnaires, recall bias may influence the results. In addition, we evaluated healthy eating behaviors using the HES-5 questionnaire, which consists of only 5 food items and does not investigate portion sizes. This tool cannot measure overall dietary intake and may cause subjective dietary assessments. However, the HES-5 is brief, straightforward, and well-correlated with the 2015 Healthy Eating Index (44). Therefore, it is a useful instrument for quickly evaluating the diet quality of participants. Finally, we did not investigate several variables that may be confounders of hypertension, such as urine indicators, potassium intake, sleep disorders, noise exposure, air pollution, family history of hypertension (28). Future studies should evaluate the impact of these factors on the relationships of hypertension.

## Conclusions

In the rural and suburban areas, people with better health literacy were less likely to have hypertension. In addition, BMI, VF, and PBF were found to be positively associated with hypertension. Notably, body fat percentage significantly mediated the association between health literacy and hypertension. The findings provide a preliminary mechanism for future research to explain the health literacy and hypertension relationship. Furthermore, health providers and policymakers should develop proper interventions to improve health literacy and control body fat for people living in rural areas, which may help to reduce the prevalence and burden of hypertension.

## Data availability statement

The raw data supporting the conclusions of this article will be made available on reasonable request to the corresponding author.

## Ethics statement

The studies involving human participants were reviewed and approved by Hanoi University of Public Health, Vietnam. The patients/participants provided their written informed consent to participate in this study.

## Author contributions

TTN, MN, YN, TTPN, MG, TT, TP, KP, KN, V-TL, C-TS, and TD: conceptualization, methodology, validation, investigation, data curation, and writing—review and editing draft. MN, TTN, and TD: formal analysis and writing—original draft. MN, TP, and TTPN: project administration. TD: supervision and funding acquisition. All authors have read and approved the final manuscript.

## Funding

This research was funded by Hai Phong University of Medicine and Pharmacy and Taipei Medical University.

## Conflict of interest

The authors declare that the research was conducted in the absence of any commercial or financial relationships that could be construed as a potential conflict of interest.

## Publisher's note

All claims expressed in this article are solely those of the authors and do not necessarily represent those of their affiliated organizations, or those of the publisher, the editors and the reviewers. Any product that may be evaluated in this article, or claim that may be made by its manufacturer, is not guaranteed or endorsed by the publisher.
