# Supplementary material for: Body mass index, body fat percentage, and visceral fat as mediators in the association between health literacy and hypertension among residents living in rural and suburban areas
Source: Front Med (Lausanne). 2022 Sep 6;9:877013. doi: 10.3389/fmed.2022.877013 (PMC9485490; doi:10.3389/fmed.2022.877013)
Supplement: Supplementary file 1 [file Table_1.DOCX]

**Body Mass Index, Body Fat Percentage, and Visceral Fat As Mediators in the Association between Health Literacy and Hypertension among Residents Living in Rural and Suburban Areas**

# Table S1. Associations of demographic characteristics, and lifestyle behaviors with hypertension among rural people (n = 1655).

| **Variables** | **Hypertension** | |
| --- | --- | --- |
|  | **OR (95% CI)** | ***p*** |
| **Age groups** |  |  |
| < 60 | 1.00 |  |
| ≥ 60 | 3.38 (2.72, 4.20) | <0.001 |
| **Gender** |  |  |
| Women | 1.00 |  |
| Men | 1.76 (1.44, 2.16) | <0.001 |
| **Ethnicity** |  |  |
| Kinh | 1.00 |  |
| Ethnic minority | 1.02 (0.84, 1.24) | 0.871 |
| **Marital status** |  |  |
| Single | 1.00 |  |
| Married | 1.45 (0.97, 2.16) | 0.071 |
| Separated/Divorced/Widowed | 3.39 (2.03, 5.68) | <0.001 |
| **Education attainment** |  |  |
| Junior high school or below | 1.00 |  |
| Senior high school | 0.47 (0.35, 0.62) | <0.001 |
| College/University or above | 0.42 (0.31, 0.57) | <0.001 |
| **Employment status** |  |  |
| Unemployed | 1.00 |  |
| Employed | 0.45 (0.36, 0.57) | <0.001 |
| **Ability to pay for healthcare** |  |  |
| Very or fairly easy | 1.00 |  |
| Very or fairly difficult | 1.83 (1.49, 2.26) | <0.001 |
| **Social status** |  |  |
| Low | 1.00 |  |
| Middle or high | 0.66 (0.54, 0.82) | <0.001 |
| **Medical conditions** |  |  |
| None | 1.00 |  |
| One or more | 1.86 (1.54, 2.27) | <0.001 |
| **Cigarret Smoking** |  |  |
| None | 1.00 |  |
| Used to | 1.85 (1.06, 3.24) | 0.030 |
| Current | 1.24 (0.96, 1.59) | 0.099 |
| **Drinking alcohol** |  |  |
| None | 1.00 |  |
| 1-3 times/month | 0.84 (0.62, 1.15) | 0.289 |
| 1-5 times/week | 1.49 (1.12, 1.98) | 0.005 |
| Everyday | 2.16 (1.39, 3.34) | 0.001 |
| **Physical activity** |  |  |
| None | 1.00 |  |
| Sometimes | 0.85 (0.61, 1.20) | 0.369 |
| Often | 0.98 (0.75, 1.28) | 0.880 |
| Everyday | 1.25 (0.98, 1.58) | 0.068 |
| **Healthy eating score**, 1-score increment | 0.98 (0.96, 1.01) | 0.148 |
| **Salt-related knowledge**, 1-score increment | 1.08 (0.96, 1.21) | 0.201 |
| **Salt-related behaviors,** 1-score increment | 0.94 (0.87, 1.01) | 0.286 |

Abbreviations: OR, odds ratio; CI, confidence interval.

# Table S2. Spearman’s correlations (rho) among the studied variables (n= 1655).

| Variables | Age | Sex | Marital status | Edu-  cation | Em-  ployment | Ability to pay | Social status | Co-  morbidity | Smoking | Drinking | PA | HES | S-Know |
| --- | --- | --- | --- | --- | --- | --- | --- | --- | --- | --- | --- | --- | --- |
| Gender | .078 |  |  |  |  |  |  |  |  |  |  |  |  |
| Marital status | .265 | -.139 |  |  |  |  |  |  |  |  |  |  |  |
| Education | -.250 | .017 | **-.303** |  |  |  |  |  |  |  |  |  |  |
| Employment | **-.418** | -.067 | -.158 | .113 |  |  |  |  |  |  |  |  |  |
| Ability to pay | .069 | -.097 | .145 | **-.359** | .029 |  |  |  |  |  |  |  |  |
| Social status | .060 | .067 | -.082 | .251 | -.119 | **-.343** |  |  |  |  |  |  |  |
| Comorbidity | .170 | .072 | .129 | -.180 | -.121 | .117 | .023 |  |  |  |  |  |  |
| Smoking | -.024 | **.415** | -.066 | -.047 | .013 | .021 | -.035 | .073 |  |  |  |  |  |
| Drinking | -.051 | **.505** | -.137 | .076 | .056 | -.121 | .044 | .002 | **.347** |  |  |  |  |
| PA | .048 | .070 | .034 | .068 | -.080 | -.045 | .058 | .077 | -.007 | .060 |  |  |  |
| HES | .031 | .002 | -.044 | .222 | .015 | -.204 | .266 | .003 | -.018 | .042 | .156 |  |  |
| S-Know | .035 | .056 | -.087 | .133 | -.047 | -.046 | .038 | .004 | .007 | .109 | .020 | .018 |  |
| S-Behave | -.058 | -.029 | -.081 | .151 | -.017 | -.003 | .063 | -.019 | -.023 | .041 | .083 | .091 | .041 |

Abbreviations: PA, physical activity; HES, healthy eating score; S-Know, salt-related knowledge; S-Behave, salt-related behaviors.

# Table S3. Health-related behaviors as mediators in the association between health literacy and hypertension (n = 1655).

| **Mediators** | **Effect** | **OR (95% CI)** | ***p* ^a^** | **% Mediated ^b^** |
| --- | --- | --- | --- | --- |
| **Cigarret smoking** | Total | 0.96 (0.95, 0.97) | <0.001 | - |
|  | Direct | 0.96 (0.95, 0.97) | <0.001 |  |
|  | Indirect | 1.00 (0.99, 1.00) | 0.761 |  |
| **Alcohol drinking** | Total | 0.96 (0.95, 0.97) | <0.001 | - |
|  | Direct | 0.96 (0.95, 0.97) | <0.001 |  |
|  | Indirect | 1.00 (0.99, 1.00) | 0.622 |  |
| **Physical activity** | Total | 0.96 (0.95, 0.97) | <0.001 | - |
|  | Direct | 0.96 (0.95, 0.98) | <0.001 |  |
|  | Indirect | 1.00 (0.99, 1.00) | 0.125 |  |
| **Healthy eating** | Total | 0.96 (0.95, 0.97) | <0.001 |  |
|  | Direct | 0.96 (0.95, 0.98) | <0.001 | - |
|  | Indirect | 1.00 (0.99, 1.00) | 0.116 |  |

Abbreviations: OR, odds ratio; CI, confidence interval.

^a^ Adjusted for age, gender, education, social status, medical conditions.

^b^ The mediated percentage was calculated only when the indirect effect was significant (*p* < 0.05).

**Table S4.** Missing data of independent variables.

| **Variables** | **Total (n = 1655)** |
| --- | --- |
|  | **n (%)** |
| **Age groups** |  |
| < 60 | 1141 |
| ≥ 60 | 514 |
| Missing | 0 |
| **Gender** |  |
| Women | 1061 |
| Men | 591 |
| Missing | 3 |
| **Ethnicity** |  |
| Kinh | 891 |
| Ethnic minorities | 764 |
| Missing | 0 |
| **Marital status** |  |
| Single | 119 |
| Married | 1378 |
| Separated/Divorced/Widowed | 140 |
| Missing | 18 |
| **Education attainment** |  |
| Junior high school or below | 1155 |
| Senior high school | 268 |
| College/University or above | 232 |
| Missing | 0 |
| **Employment status** |  |
| Unemployed | 383 |
| Employed | 1272 |
| Missing | 0 |
| **Ability to pay for healthcare** |  |
| Very or fairly easy | 591 |
| Very or fairly difficult | 1056 |
| Missing | 8 |
| **Social status** |  |
| Low | 480 |
| Middle or high | 1166 |
| Missing | 7 |
| **Medical conditions** |  |
| None | 843 |
| One or more | 811 |
| Missing | 1 |
| **Cigarette smoking** |  |
| None | 1299 |
| Used to | 52 |
| Current | 302 |
| Missing | 2 |
| **Drinking alcohol** |  |
| None | 1120 (67.8) |
| 1-3 times/month | 203 (12.3) |
| 1-5 times/week | 239 (14.5) |
| Everyday | 90 (5.4) |
| Missing | 3 |
| **Physical activity** |  |
| None | 685 (41.4) |
| Sometimes | 175 (10.6) |
| Often | 322 (19.5) |
| Everyday | 471 (28.5) |
| Missing | 2 |
| **BMI, kg/m2** |  |
| Underweight | 204 (12.4) |
| Normal weight | 929 (73.3) |
| Overweight/obese | 512 (14.3) |
| Missing | 10 |
| **Abdominal obesity** |  |
| No | 813 (49.1) |
| Yes | 842 (50.9) |
| Missing | 0 |
| **Visceral fat level** |  |
| Normal | 1445 (87.5) |
| High | 207 (12.5) |
| Missing | 3 |
| **Body fat percentage** |  |
| Low | 145 (8.8) |
| Normal | 625 (38.0) |
| High | 873 (53.1) |
| Missing | 12 |
| **Healthy eating score**, mean±SD | 9.5 ± 4.5 |
| Missing | 0 |
| **Salt-related knowledge,** mean±SD | 1.2 ± 0.8 |
| Missing | 0 |
| **Salt-related behaviors,** mean±SD | 11.2 ± 2.0 |
| Missing | 937 |
| **Health literacy**, mean±SD | 22.1 ± 11.2 |
| Missing | 0 |
